# Supplementary material for: CEBPA restricts alveolar type 2 cell plasticity during development and injury-repair
Source: Res Sq. 2023 Dec 14:rs.3.rs-3521387. Preprint. [Version 1] doi: 10.21203/rs.3.rs-3521387/v1 (PMC10760240; doi:10.21203/rs.3.rs-3521387/v1)
Supplement: Supplement 1 [file NIHPPrs3521387v1-supplement-1.pdf]

SUPPLEMENTARY FIGURES

Fig. S1. Time-course analysis of AT2 cell development and CEBPA expression

(**A**) Biological process GO terms for the nearest genes of the 4 ATAC-seq clusters in Fig. 1D. (**B**) Heatmap of ChromVAR deviation scores to the 100 most variable motifs across time. (**C**) Monocle pseudotemporal expression changes of 5 CEBP family members across 12 time points in Fig. 1B. *Cebpa*, but not other CEBP genes, reaches maximal expression upon AT2 specification. *Cebpe* is excluded due to lack of expression in alveolar epithelial cells. (**D**) Feature plots of Fig. 1A showing robust expression of *Cebpa*, but not other CEBP genes, in nascent AT2 cells. (**E**) Confocal images showing CEBPA is not expressed in SOX9 progenitors nor HOPX+ AT1 cells as SOX9 progenitors differentiate into AT1 and AT2 cells from E16.5 to E18.5. (**F**) Confocal images showing CEBPA is expressed in LAMP3+ AT2 cells but not HOPX+ AT1 cells. Scale: 10 um. See Table S1 for raw data.

Fig. S2. Characterization of neonatal *Cebpa* mutant AT2 cells

(**A**) Confocal images showing loss of CEBPA and IL33 in GFP+ recombined neonatal mutant AT2 cells (filled vs open arrowhead). AM, alveolar macrophage; *, escaper of *Cebpa* deletion still expressing IL33. (**B**) Stitched TEM images showing higher cell density in the mutant. Quantification of lamellar bodies for Fig. 2C (Student's t-test). (**C**) Confocal images showing adjoining (ECAD) ectopic SOX9 cells in the mutant, resembling SOX9 progenitors at embryonic branch tips. Scale: 10 um. See Table S2 for raw data.

Fig. S3. Multiome and staining of neonatal *Cebpa* mutant AT2 cells

(**A**) FACS gating strategy to purify lung epithelial cells. (**B**) Split feature plots of Fig. 3C to better visualize the control and mutant. (**C**) Confocal images showing that HOPX+ mutant AT2 cells do not express LAMP3 (arrowhead). (**D**) Confocal images showing persistent, albeit somewhat lower, SFTPC in mutant AT2 cells. (**E**) Violin plots of control and mutant AT2 cells in Fig. 3A showing a small decrease in surfactant gene expression. (**F**) Confocal images showing normal NKX2-1 expression in mutant AT2 cells. Scale: 10 um.

Fig. S4. ChIP-seq and comparison of neonatal vs mature *Cebpa* mutant AT2 cells

(**A**) FACS gating strategy to purify AT2 nuclei for ChIP-seq. (**B**) Coverage plots showing a putative regulatory region 3' to *Sox9* (box) that opens with more NKX2-1 binding upon *Cebpa* deletion, gradually closes and loses NKX2-1 binding during AT2 cell development in wild type lungs, and does not have CEBPA binding. (**C**) Confocal images showing loss of CEBPA and IL33 in GFP+ recombined mature mutant AT2 cells, except for escapers of deletion (asterisk). Scale: 10 um. (**D**) Quantification of lamellar bodies in mature AT2 cells for Fig. 5B (Student's t-test). (**E**) Feature plots of motif activities for Fig. 5C. (**F**) Top: Venn diagram comparison of increased peaks in neonatal (Fig. 3F) vs mature (Fig. 5G) mutant AT2 cells. Bottom: heatmaps and profile plots showing that neonatal specific increased peaks gradually lose accessibility (log2 fold change) from E16.5 to 7-wk.

Fig. S5. Characterization of control and *Cebpa* mutant lungs exposed to Sendai virus or saline

(**A**) Confocal images showing SOX9 reactivation, distinct from KRT8 expression, near lobe edges (I) and airways/macro-vessels (II, III). Scale: 100 um (10 um for insets). (**B**) Confocal images showing no SOX9 reactivation, HOPX expression, nor high KRT8 expression upon saline administration in control and mutant lungs. Baseline KRT8 expression is present in all AT2 cells. Scale: 10 um. (**C**) Confocal images showing that KRT8/CLDN4+ cells have low LAMP3 and no HOPX (arrowhead). Scale: 10 um. (**D**) Confocal images showing that AT1-like cells expressing HOPX (arrowhead) are no longer cuboidal (ECAD). Scale: 10 um.

Fig. S6. Additional characterization of SOX9 and CEBPA in infected control and *Cebpa* mutant lungs

(**A**) Confocal images showing that reactivated SOX9 in infected mutant AT2 cells is not in KRT8/CLDN4+ cells (yellow arrowhead) nor AT1-like cells (HOPX+; white arrowhead). Scale: 10 um. (**B**) Confocal images showing loss of CEBPA in KRT8/CLDN+ cells (left) and AT1-like cells (HOPX+; arrowhead) (right) even in the control lung. Open arrowhead, low CEBPA. Scale: 10 um. (**C**) Feature plots for Fig. 6G showing loss/reduction of *Cebpa* and *Lamp3* in KRT8/CLDN4+ and AT1-like cells even in the control lung.

SUPPLEMENTARY TABLES

Table S1. Raw data for Fig. 1, S1.

Table S2. Raw data for Fig. 2, S2.

Table S3. Raw data for Fig. 3.

Table S4. Raw data for Fig. 4.

Table S5. Raw data for Fig. 5.

Table S6. Raw data for Fig. 6.

SUPPLEMENTARY FILE 1: Scripts for genomic data analysis.

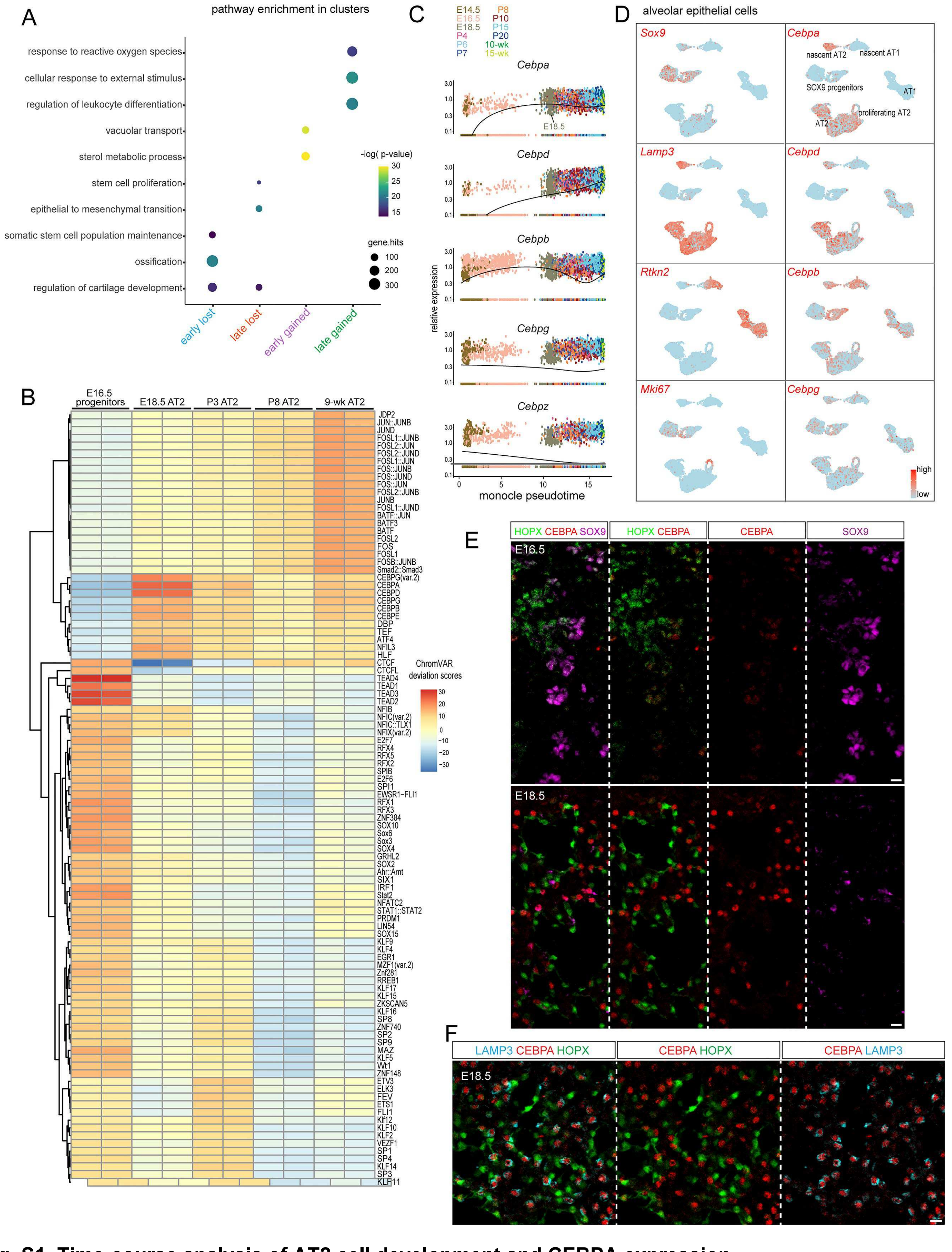

Fig. S1. Time-course analysis of AT2 cell development and CEBPA expression

(**A**) Biological process GO terms for the nearest genes of the 4 ATAC-seq clusters in Fig. 1D. (**B**) Heatmap of ChromVAR deviation scores to the 100 most variable motifs across time. (**C**) Monocle pseudotemporal expression changes of 5 CEBP family members across 12 time points in Fig. 1B. *Cebpa*, but not other CEBP genes, reaches maximal expression upon AT2 specification. *Cebpe* is excluded due to lack of expression in alveolar epithelial cells. (**D**) Feature plots of Fig. 1A showing robust expression of *Cebpa*, but not other CEBP genes, in nascent AT2 cells. (**E**) Confocal images showing CEBPA is not expressed in SOX9 progenitors nor HOPX+ AT1 cells as SOX9 progenitors differentiate into AT1 and AT2 cells from E16.5 to E18.5. (**F**) Confocal images showing CEBPA is expressed in LAMP3+ AT2 cells but not HOPX+ AT1 cells. Scale: 10 um. See Table S1 for raw data.

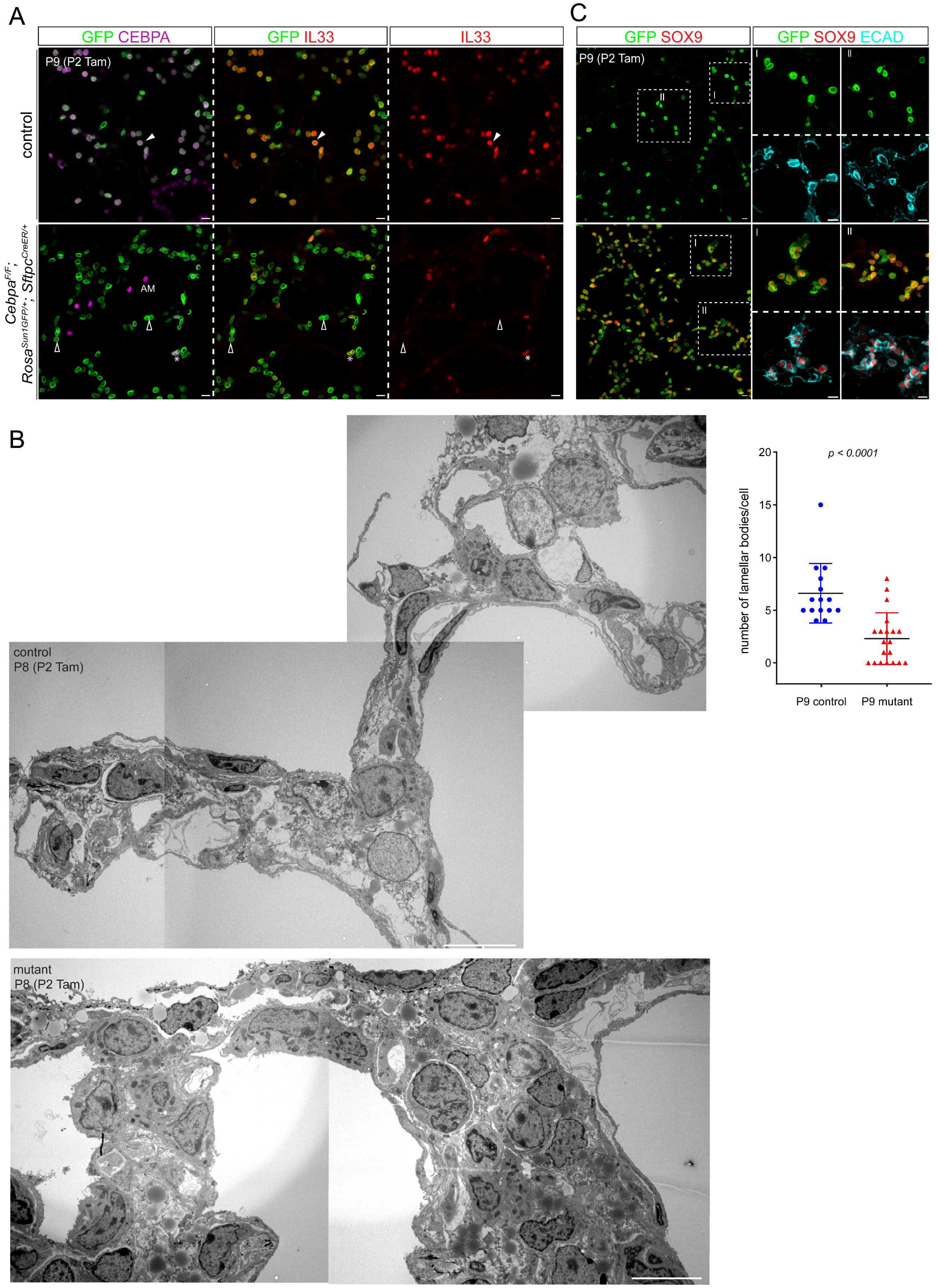

Fig. S2. Characterization of neonatal *Cebpa* mutant AT2 cells

(**A**) Confocal images showing loss of CEBPA and IL33 in GFP+ recombined neonatal mutant AT2 cells (filled vs open arrowhead). AM, alveolar macrophage; *, escaper of *Cebpa* deletion still expressing IL33. (**B**) Stitched TEM images showing higher cell density in the mutant. Quantification of lamellar bodies for Fig. 2C (Student's t-test). (**C**) Confocal images showing adjoining (ECAD) ectopic SOX9 cells in the mutant, resembling SOX9 progenitors at embryonic branch tips. Scale: 10 um. See Table S2 for raw data.

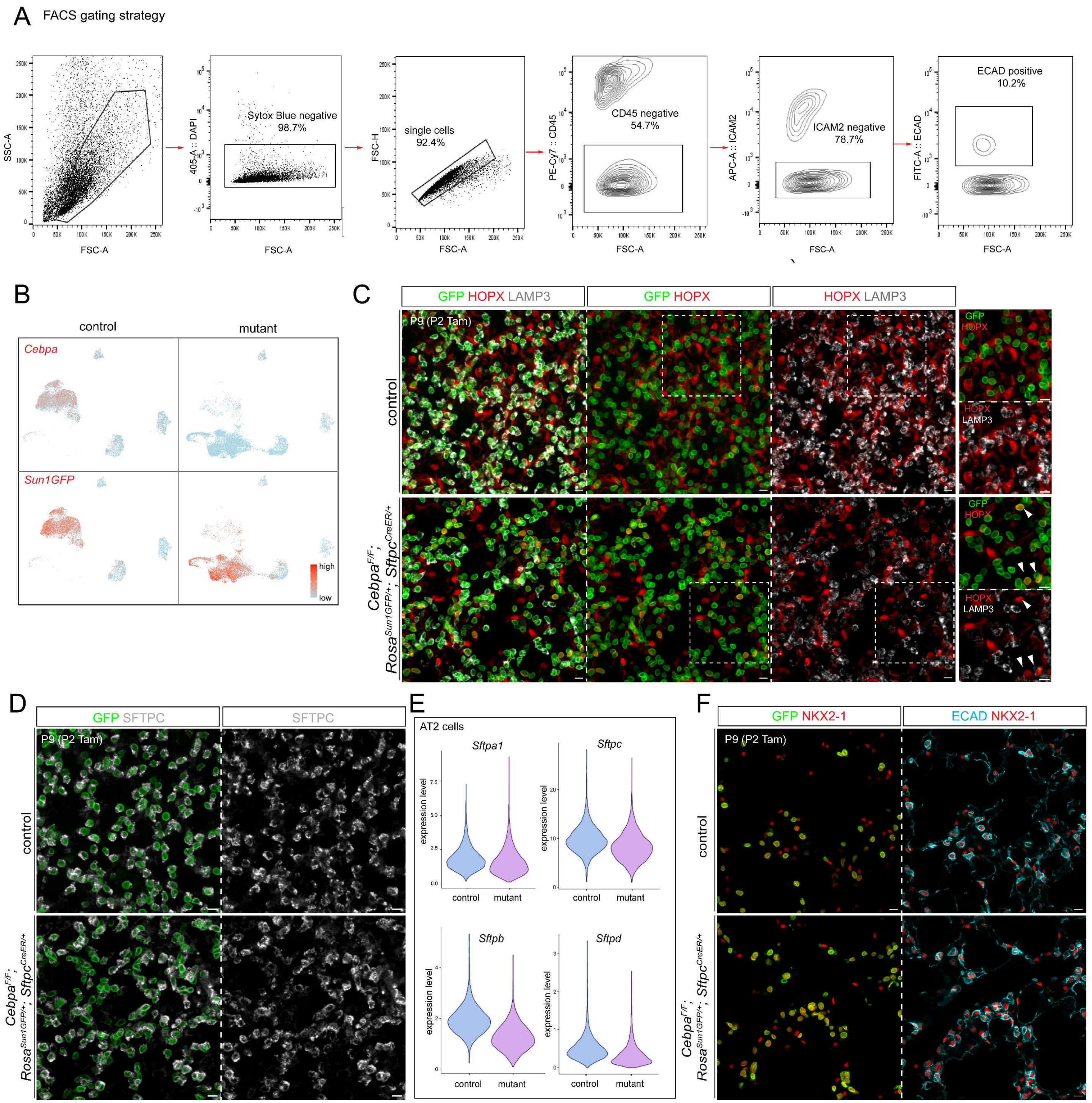

Fig. S3. Multiome and staining of neonatal *Cebpa* mutant AT2 cells

(**A**) FACS gating strategy to purify lung epithelial cells. (**B**) Split feature plots of Fig. 3C to better visualize the control and mutant. (**C**) Confocal images showing that HOPX+ mutant AT2 cells do not express LAMP3 (arrowhead). (**D**) Confocal images showing persistent, albeit somewhat lower, SFTPC in mutant AT2 cells. (**E**) Violin plots of control and mutant AT2 cells in Fig. 3A showing a small decrease in surfactant gene expression. (**F**) Confocal images showing normal NKX2-1 expression in mutant AT2 cells. Scale: 10 um.

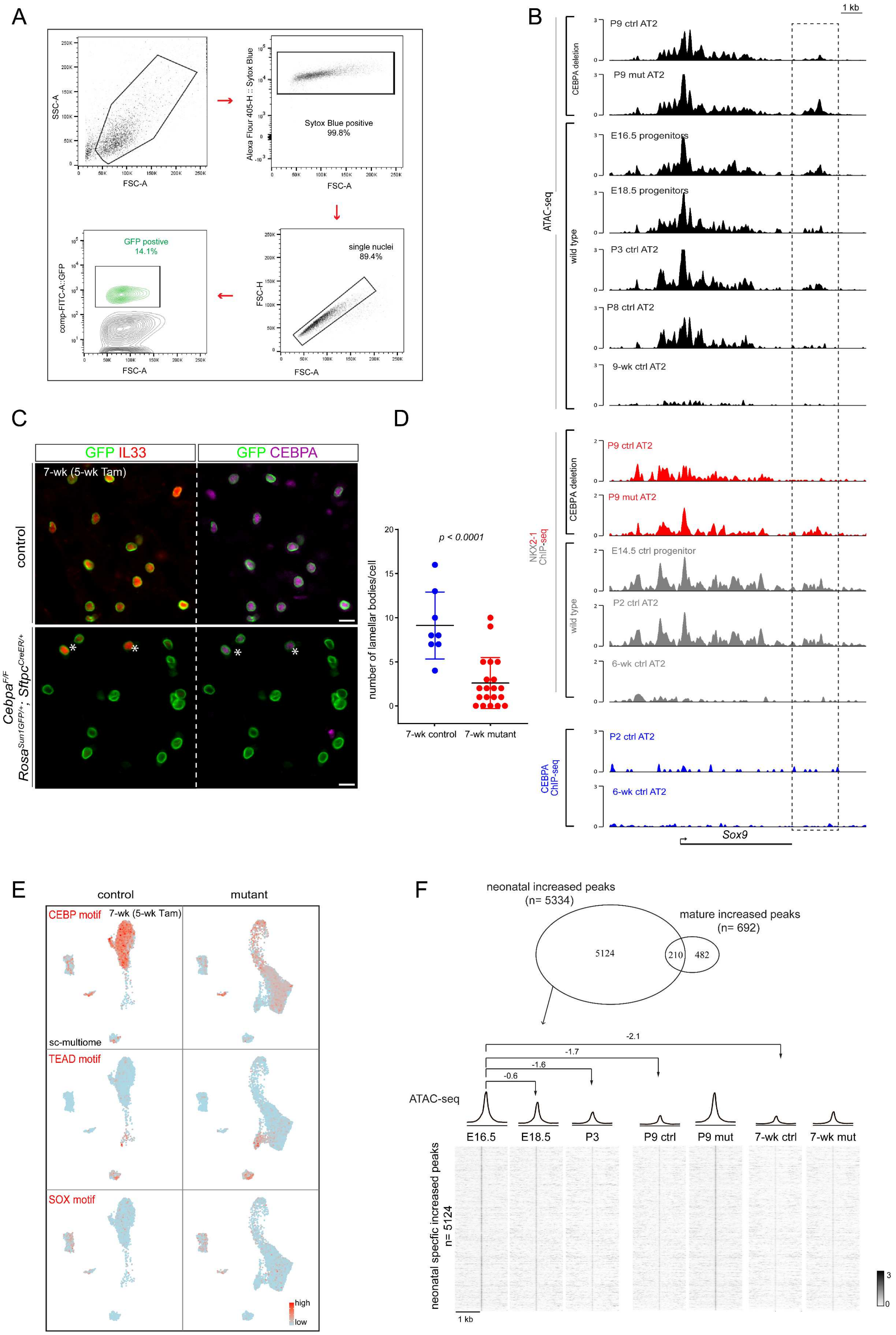

A
SSC-A
FSC-A
Alexa Flour 405-H :: Sytox Blue
Sytox Blue positive
99.8%
FSC-H
single nuclei
89.4%
comp-FITC-A::GFP
GFP postive
14.1%
B
1 kb
ATAC-seq
CEBPA deletion
P9 ctrl AT2
P9 mut AT2
wild type
E16.5 progenitors
E18.5 progenitors
P3 ctrl AT2
P8 ctrl AT2
9-wk ctrl AT2
NKX2-1 ChIP-seq
E14.5 ctrl progenitor
P2 ctrl AT2
6-wk ctrl AT2
CEBPA ChIP-seq
Sox9
C
GFP IL33
GFP CEBPA
7-wk (5-wk Tam)
control
Cebpa F/F; Rosa Sun1GFP/+; Sftpc CreER/+
D
p < 0.0001
number of lamellar bodies/cell
7-wk control
7-wk mutant
E
control
mutant
CEBP motif
7-wk (5-wk Tam)
sc-multiome
TEAD motif
SOX motif
high
low
F
neonatal increased peaks
(n= 5334)
mature increased peaks
(n= 692)
5124
210
482
-2.1
-1.7
-1.6
-0.6
ATAC-seq
E16.5
E18.5
P3
P9 ctrl
P9 mut
7-wk ctrl
7-wk mut
neonatal specific increased peaks
n= 5124
1 kb

Fig. S4. ChIP-seq and comparison of neonatal vs mature *Cebpa* mutant AT2 cells

(**A**) FACS gating strategy to purify AT2 nuclei for ChIP-seq. (**B**) Coverage plots showing a putative regulatory region 3' to *Sox9* (box) that opens with more NKX2-1 binding upon *Cebpa* deletion, gradually closes and loses NKX2-1 binding during AT2 cell development in wild type lungs, and does not have CEBPA binding. (**C**) Confocal images showing loss of CEBPA and IL33 in GFP+ recombined mature mutant AT2 cells, except for escapers of deletion (asterisk). Scale: 10 um. (**D**) Quantification of lamellar bodies in mature AT2 cells for Fig. 5B (Student's t-test). (**E**) Feature plots of motif activities for Fig. 5C. (**F**) Top: Venn diagram comparison of increased peaks in neonatal (Fig. 3F) vs mature (Fig. 5G) mutant AT2 cells. Bottom: heatmaps and profile plots showing that neonatal specific increased peaks gradually lose accessibility (log2 fold change) from E16.5 to 7-wk.

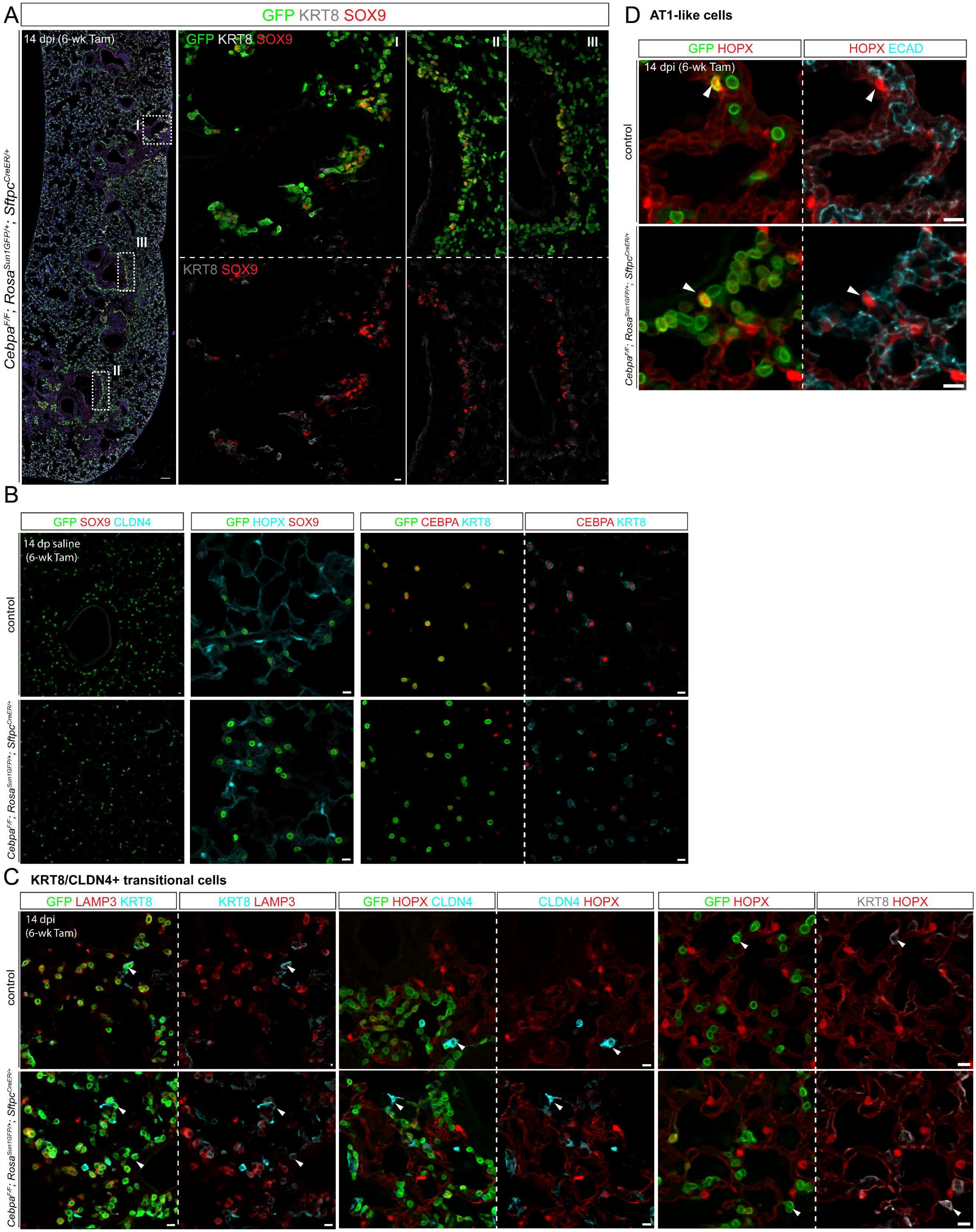

Fig. S5. Characterization of control and *Cebpa* mutant lungs exposed to Sendai virus or saline

(**A**)Confocal images showing SOX9 reactivation, distinct from KRT8 expression, near lobe edges (I) and airways/macro-vessels (II, III). Scale: 100 um (10 um for insets). (**B**) Confocal images showing no SOX9 reactivation, HOPX expression, nor high KRT8 expression upon saline administration in control and mutant lungs. Baseline KRT8 expression is present in all AT2 cells. Scale: 10 um. (**C**) Confocal images showing that KRT8/CLDN4+ cells have low LAMP3 and no HOPX (arrowhead). Scale: 10 um. (**D**) Confocal images showing that AT1-like cells expressing HOPX (arrowhead) are no longer cuboidal (ECAD). Scale: 10 um.

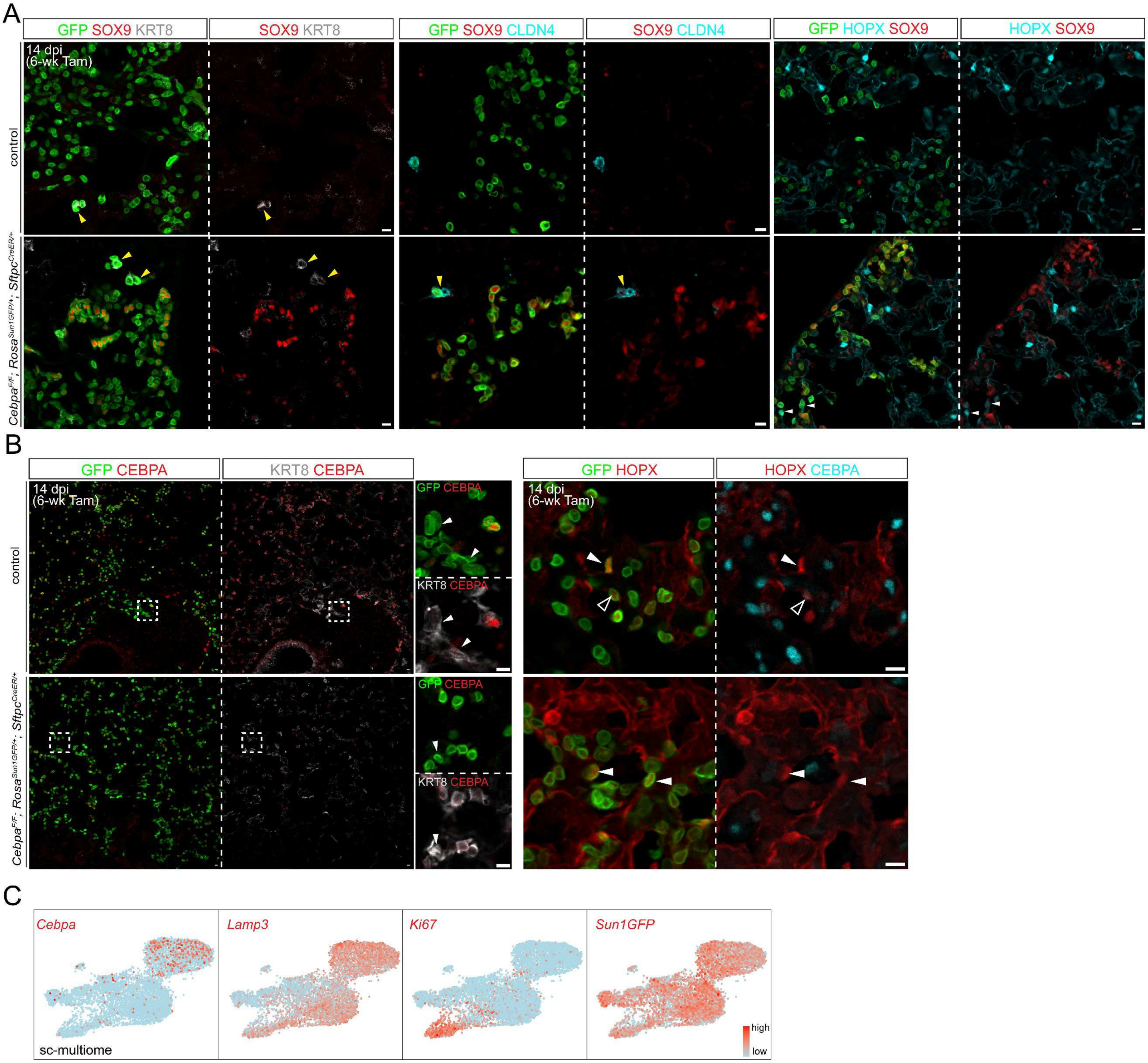

Fig. S6. Additional characterization of SOX9 and CEBPA in infected control and *Cebpa* mutant lungs

(**A**) Confocal images showing that reactivated SOX9 in infected mutant AT2 cells is not in KRT8/CLDN4+ cells (yellow arrowhead) nor AT1-like cells (HOPX+; white arrowhead). Scale: 10 um. (**B**) Confocal images showing loss of CEBPA in KRT8/CLDN+ cells (left) and AT1-like cells (HOPX+; arrowhead) (right) even in the control lung. Open arrowhead, low CEBPA. Scale: 10 um. (**C**) Feature plots for Fig. 6G showing loss/reduction of *Cebpa* and *Lamp3* in KRT8/CLDN4+ and AT1-like cells even in the control lung.

Supplementary Files

This is a list of supplementary files associated with this preprint. Click to download.

- SUPPLEMENTARYTABLES.zip
- SUPPLEMENTARYFILE1.txt
